# SPS1 deficiency-triggered *PGRP-LC* and *Toll* expression controls innate immunity in *Drosophila* S2 cells

**DOI:** 10.1242/bio.059295

**Published:** 2022-08-01

**Authors:** Tack-Jin Yoo, Myoung Sup Shim, Jeyoung Bang, Jin-Hong Kim, Byeong Jae Lee

**Affiliations:** 1 Department of Biological Sciences, College of Natural Sciences, Seoul National University, Seoul 08826, Korea; 2 Department of Ophthalmology, Duke Eye Center, Duke Eye Center, Duke University, Durham, NC 27705, USA

**Keywords:** SPS1, Innate immune system, IMD pathway, Toll pathway, PGRP-LC, Toll

## Abstract

Selenophosphate synthetase 1 (SPS1) is an essential gene for the cell growth and embryogenesis in *Drosophila melanogaster*. We have previously reported that SPS1 deficiency stimulates the expression of genes responsible for the innate immune system, including antimicrobial peptides (AMPs), in *Drosophila* S2 cells. However, the underlying mechanism has not been elucidated. Here, we investigated the immune pathways that control the SPS1-deficiency-induced expression of AMPs in S2 cells. It was found that the activation of AMP expression is regulated by both immune deficiency (IMD) and the Toll pathway. Double knockdown of the upstream genes of each pathway with SPS1 showed that the peptidoglycan recognition protein-LC (PGRP-LC) and Toll genes are targeted by SPS1 for regulating these pathways. We also found that the IMD and Toll pathway regulate AMP expression by cross-talking. The levels of *PGRP-LC* and *Toll* mRNAs were upregulated upon *Sps1* knockdown (6.4±0.36 and 3.2±0.45-fold, respectively, *n*=3). Overexpression of each protein also upregulated AMPs. Interestingly, PGRP-LC overexpression upregulated AMP more than Toll overexpression. These data strongly suggest that SPS1 controls the innate immune system of *D. melanogaster* through regulating *PGRP-LC* and *Toll* expression.

## Abstract (precis)

**Summary:** This study provides insights into the function of SEPHS1 regulating the innate immune system of *Drosophila* through controlling PGRP-LC and Toll transcription even without infection.

## INTRODUCTION

Selenium is an essential trace element in the diet of humans and many other life forms. An appropriate amount of selenium offers many health benefits, such as preventing cancer and heart disease, acting as an antiviral agent, scavenging reactive oxygen species (ROS), and augmenting the immune system and male reproduction (Brigelius-Flohé and Sies, 2016; Gladyshev et al., 2016; Na et al., 2018). The many benefits of selenium may be due to its existence in selenoproteins in the form of the amino acid selenocysteine (Sec) (Hatfield and Gladyshev, 2002; Brigelius-Flohe, 2008; Lu and Holmgren, 2009). Sec is the 21st amino acid and enters into a growing peptide in response to the UGA codon during translation (Lee et al., 1989; Leinfelder et al., 1989; Longtin, 2004; Squires and Berry, 2008; Allmang et al., 2009). The active donor of selenium in Sec biosynthesis is monoselenophosphate (Glass et al., 1993). It is synthesized from selenide and ATP by the enzyme selenophosphate synthetase (SPS, also called SelD or patufet) (Ehrenreich et al., 1992). Only one type of SPS exists in prokaryotes, including Archaea. However, there are two isoforms of SPS, SPS1 and 2, in eukaryotes (Guimaraes et al., 1996). In higher animals, such as mammals, SPS is referred to as SEPHS, because sucrose-phosphate synthase is also designated as SPS. However, there is no sucrose–phosphate synthase in *Drosophila melanogaster*. The amino acid sequences of SPS1 and 2 are highly conserved. One of the main differences between the sequences of SPS1 and 2 is that they have an arginine and Sec, respectively, in a homologous region (Low et al., 1995). Additionally, only SPS2 has selenophosphate synthesis activity (Xu et al., 2007).

In *D. melanogaster*, SPS1 deficiency leads to aberrant imaginal-disc morphology and embryonic lethality (Alsina et al., 1998). Furthermore, *Sps1* knockdown decelerates cell growth, activates the innate immunity by upregulating AMPs, increases ROS levels, and induces megamitochondria formation in *Drosophila* S2 cells (Shim et al., 2009; Lee et al., 2011). These phenotypes are caused through downregulation of pyridoxal phosphate (PLP), a biologically active form of vitamin B6 (Lee et al., 2011). In mice, systemic knockout of *Sephs1* (*Sps1*) gradually increases the oxidative stress, thereby impairing gastrulation-related signaling pathways and causing embryonic lethality (Tobe et al., 2016; Bang et al., 2021). In 2H11 cells, SEPHS1 deficiency increases the superoxide level, thereby causing DNA damage, which suppresses the cell proliferation and impairs the cell functions (Jung et al., 2021).

Innate immunity is an important defense system against infections in metazoans (Janeway and Medzhitov, 2002; Lemaitre and Hoffmann, 2007). As with all invertebrates, *D. melanogaster* depends entirely on innate immunity to thwart infections (Lemaitre and Hoffmann, 2007). Antimicrobial peptides (AMPs) are one of the main effector molecules in the innate immune system. AMPs protect the host by destroying the cell wall of invading microorganisms with cationic and amphipathic peptides (Zhang and Gallo, 2016). The induction of AMP production upon infection is regulated via two distinct signaling pathways – the Toll and IMD pathways – in *D. melanogaster* (Lemaitre and Hoffmann, 2007; Valanne et al., 2011; Myllymaki et al., 2014). The Toll pathway is used for activating the expression of *Drosomycin* (*Drs*) and *Metchnikowin* (*Mtk*), and these AMPs are required to protect cells from infections by fungi or Gram-positive bacteria. The active form of spätzle (Spz), cleaved by the spätzle-processing enzyme (SPE), activates the Toll signaling (Kanoh et al., 2015) and finally induces the nuclear translocation of the proteins nuclear factor-kappa B (NF-κB), dorsal-related immunity factor (DIF), and dorsal, thereby activating the expression of AMP genes, including *Drs* and *Mtk* (Lindsay and Wasserman, 2014). The IMD pathway is activated upon detecting diaminopimelic acid (DAP)-type peptidoglycans, which are derived from Gram-negative bacteria, via the transmembrane receptor PGRP-LC (Kleino and Silverman, 2014). This transmembrane receptor transduces the signal to downstream factors, including the adaptor proteins (IMD) and NF-κB (relish), and eventually AMPs, such as Drosocin (Dro), Diptericin (Dpt), Attacin (Att), and Cecropin (Cec), are upregulated (Paquette et al., 2010).

Although SPS1 is involved in the regulation of the *D. melanogaster* innate immune system, the mechanisms whereby SPS1 regulates the AMP production are elusive. In this study, we investigated the signaling components through which SPS1 modulates *D. melanogaster* innate immunity and found that *PGRP-LC* and *Toll*, two genes of transmembrane receptors in the IMD and Toll pathways, respectively, are the primary targets in SPS1-deficiency–induced AMP production.

## RESULTS

### SPS1 deficiency activates innate immunity – the IMD and Toll pathways

**Fig. 1.**
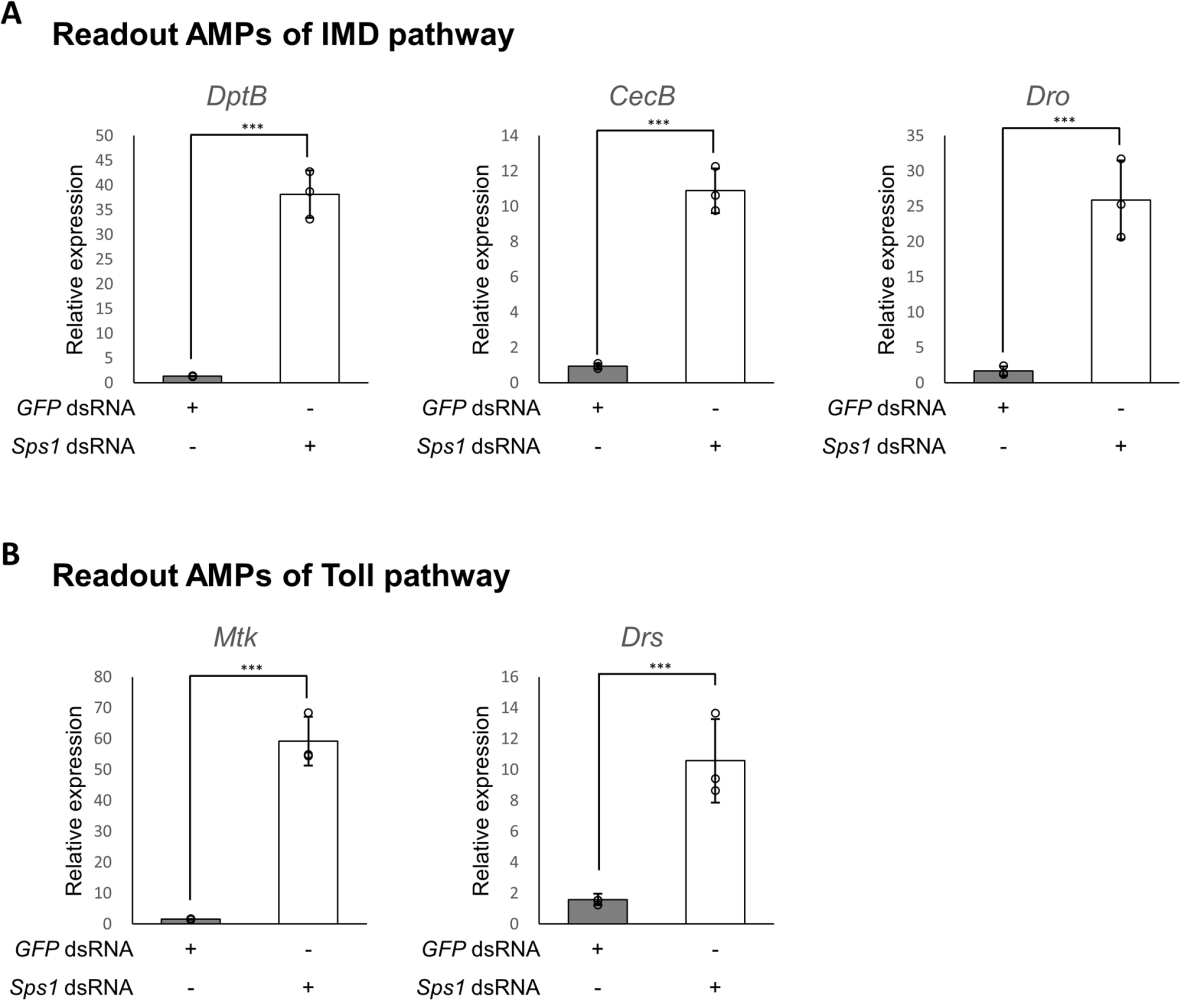
**SPS1 deficiency upregulates the AMPs of the IMD and Toll pathways.** Five days after adding the *Sps1* dsRNA, mRNA levels were measured via RT-qPCR using rp49 as a control for normalization. The y-axis represents the relative mRNA level of each gene in the cells treated with the *Sps1* dsRNA to that of no-dsRNA-treated cells. (A) Readout AMPs of the IMD pathway. (B) Readout AMPs of the Toll pathway. *** indicates *P*-value <0.001, based on unpaired Student's *t*-test. *DptB*, *Diptericin B*; *CecB*, *Cecropin B*; *Dro*, *Drosocin*; *Mtk*, *Metchnikowin*; *Drs*, *Drosomycin*; *GFP, green fluorescent protein*.

We have previously reported that *Sps1* knockdown upregulates AMPs that are responsible for *D. melanogaster* innate immunity (Lee et al., 2011). To elucidate which immune pathways are regulated upon SPS1 deficiency, *Sps1* was knocked down in S2 cells, and the expression levels of AMPs were measured using RT-qPCR after 5 days. *DptB*, *CecB*, *Dro*, *Mtk*, and *Drs*, which are AMP genes widely used to assess whether the innate immune system is activated, were selected as AMP markers. Data analysis revealed that AMPs of both the IMD (*DptB*, *CecB*, *and Dro*) and Toll (*Mtk* and *Drs*) pathways were upregulated 10–50 fold (*P*<0.001) in SPS1-deficient cells (Fig. 1A and B). These results indicate that SPS1 deficiency activates both the IMD and Toll pathways.

### SPS1 regulates innate immunity by targeting the transmembrane receptors PGRP-LC and Toll

To identify the target genes through which SPS1 regulates the expression of the AMPs in the IMD and Toll pathways, the upstream genes in each pathway were individually knocked down along with *Sps1*.

**Fig. 2.**
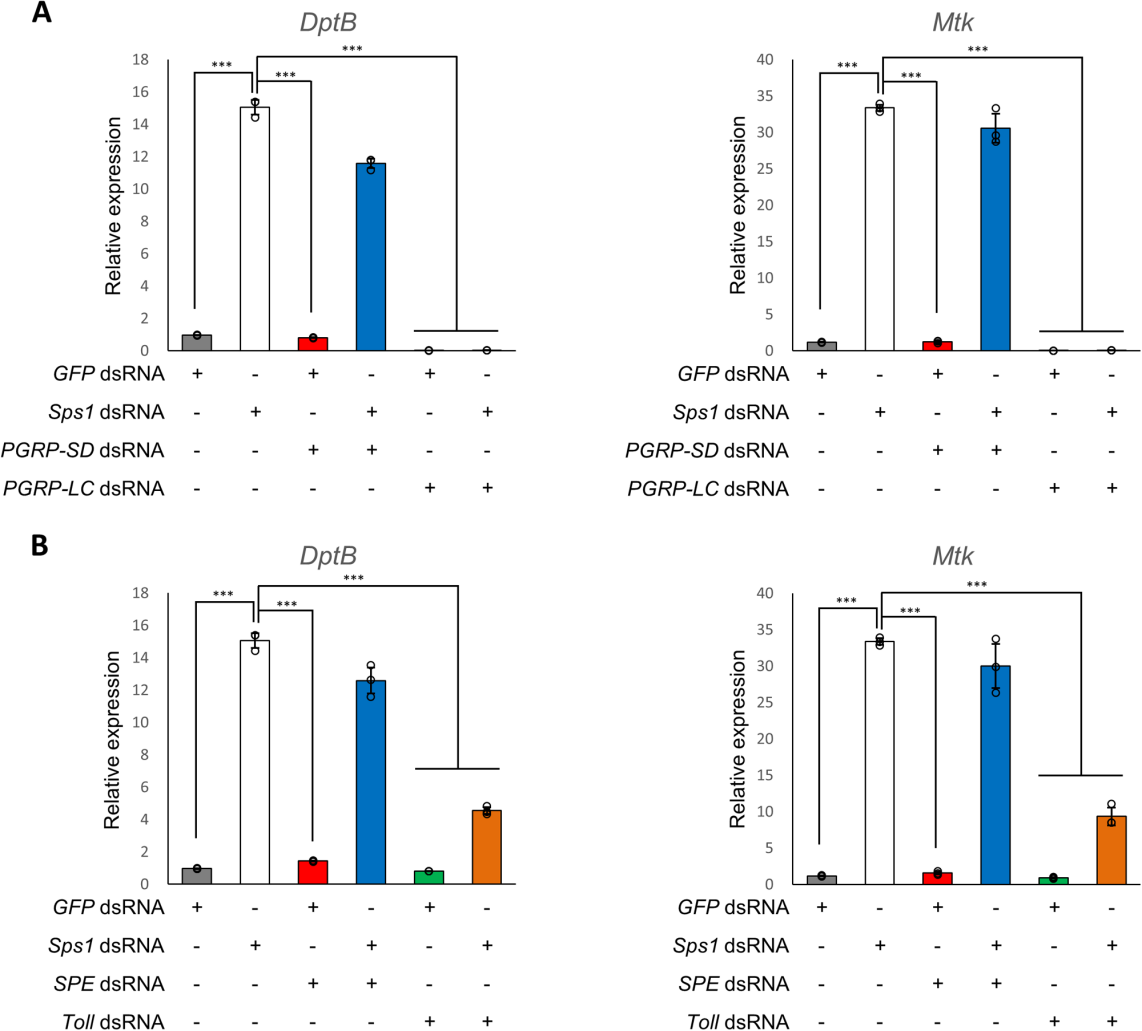
**SPS1 deficiency upregulates the AMPs of the IMD and Toll pathways through the transmembrane receptors PGRP-LC and Toll, respectively.** Five days after adding each dsRNA with *Sps1* dsRNA, the mRNA levels of AMP genes were measured via RT-qPCR using rp49 as a control for normalization. Relative expression levels of AMP genes after knocking down *Sps1* alongside *PGRP-SD* or *PGRP-LC* (A), and alongside *SPE* or *Toll* (B). *** indicates *P*-value <0.001, based on one-way ANOVA with Tukey's multiple comparison test. *Mtk*, *Metchnikowin*; *DptB*, *Diptericin B*; *GFP, green fluorescent protein*.

First, to investigate the IMD pathway, *PGRP-SD*, the most upstream gene in the IMD pathway and *Sps1* were co-knocked down. As shown in Fig. 2A, *DptB* and *Mtk* were upregulated upon *Sps1* knockdown were not recovered upon *PGRP-SD/Sps1* co-knockdown. However, intriguingly, the expression levels of *DptB* and *Mtk* were significantly reduced to the background levels (GFP control) upon *PGRP-LC*/*Sps1* co-knockdown (*P*<0.001) (Fig. 2A). Other AMP genes (*CecB*, *Dro*, and *Drs*) showed similar results (Fig. S1A). These results indicate that SPS1 deficiency affects the IMD pathway by regulating PGRP-LC.

Next, the Toll pathway was likewise investigated. No recovery was observed when *SPE* and *Sps1* were co-knocked down (Fig. 2B). However, when *Toll* and *Sps1* were co-knocked down, the expression of *DptB* and *Mtk* was decreased significantly (*P*<0.001, Fig. 2C). Other AMP genes (*CecB*, *Dro*, and *Drs*) also showed similar results (Fig. S1B). The expression data for all the AMP genes analyzed in this study are summarized in Table 1. The knockdown efficiency was >90% for all the genes (Fig. S1C). Interestingly, the readout AMPs of the Toll pathway were downregulated upon co-knocking down *PGRP-LC*, which participates in the IMD pathway, and vice versa, (the readout AMPs of IMD pathway were also downregulated by the co-knocking down *Toll*). These data suggest that there is a crosstalk between the IMD and Toll pathways (Fig. 2). Taken together, it can be concluded that SPS1 regulates AMP expression through a crosstalk between the IMD and Toll pathways at the transmembrane receptor level, namely through *PGRP-LC* and *Toll*, respectively.

**Table 1.**
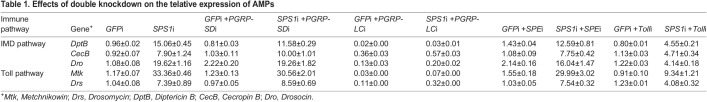
Effects of double knockdown on the telative expression of AMPs

### SPS1 regulates the transcription of PGRP-LC and Toll

**Fig. 3.**
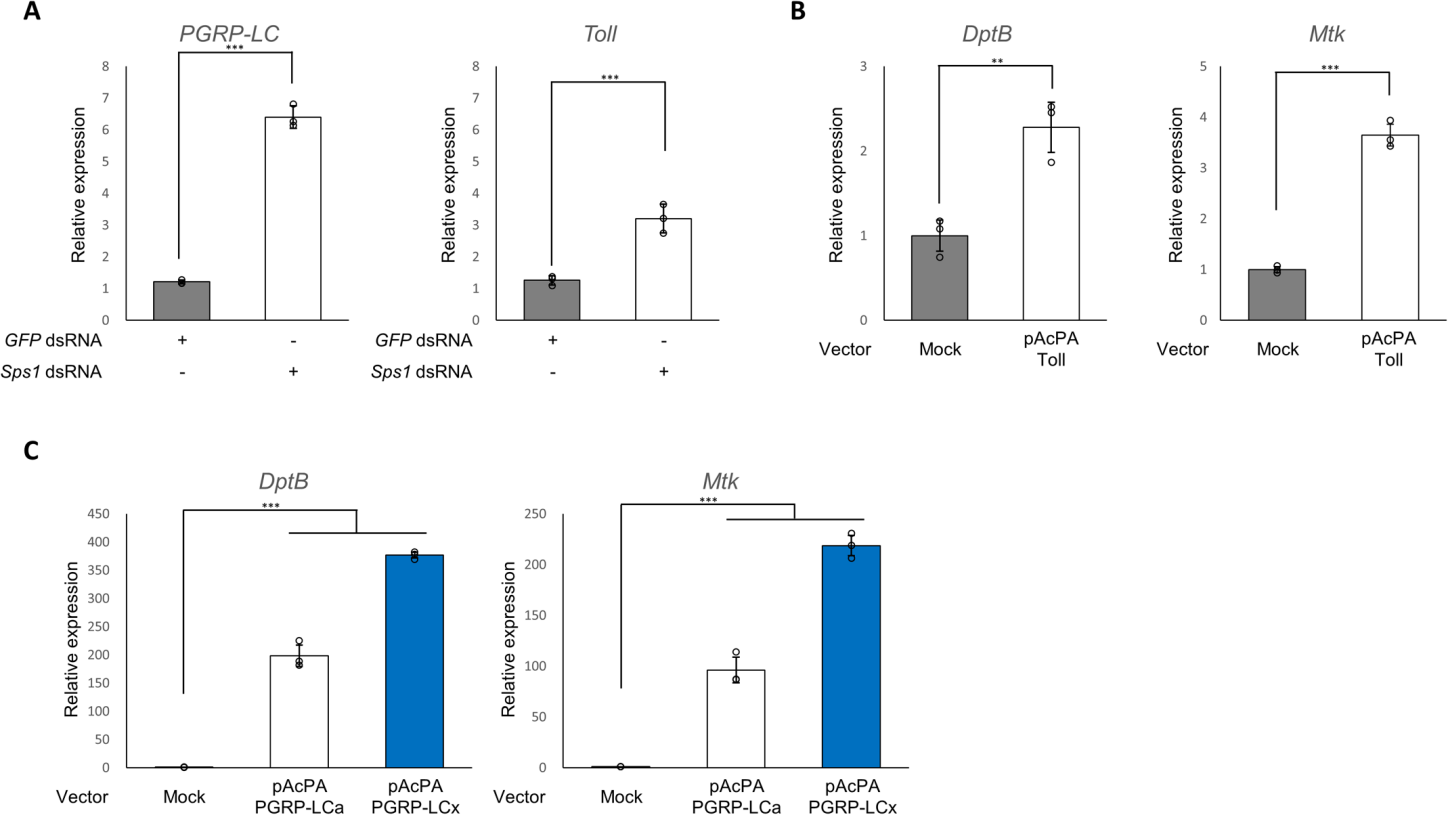
**SPS1 deficiency upregulates *PGRP-LC* and *Toll*, and overexpression of *PGRP-LC* or *Toll* induces AMP expression.** (A) Five days after adding the *Sps1* dsRNA, the mRNA levels of each gene were measured via RT-qPCR using rp49 for normalization. (B,C) Three days after transfection of S2 cells with pAcPA-PGRP-LCa, pAcPA-PGRP-LCx, or pAcPA-Toll, the mRNA levels of AMP genes were likewise measured. ** and *** indicate *P*-values <0.01 and <0.001, respectively, based on unpaired Student's *t*-test. *Mtk*, *Metchnikowin*; *DptB*, *Diptericin B*; *GFP, green fluorescent protein*.

Since PGRP-LC and Toll are the targets of SPS1, and it has been reported that ecdysone regulates the innate immune system by upregulating *PGRP-LC* (Rus et al., 2013), we hypothesized that SPS1 deficiency upregulates *PGRP-LC* and *Toll*. As shown in Fig. 3A, *Sps1* knockdown significantly increased the levels of the *PGRP-LC* and *Toll* mRNAs (6.4±0.36 and 3.2±0.45-fold, respectively, *n*=3), indicating that SPS1 regulates both *PGRP-LC* and *Toll* presumably at the transcription level. Interestingly, SPS1 deficiency upregulated *PGRP-LC* by approximately 2-fold (*P*<0.001, *n*=3) compared with that of *Toll*, suggesting that SPS1 deficiency has a stronger effect on PGRP-LC than on Toll.

### Increased expression of PGRP-LC or Toll activates the innate immune system

To decipher whether upregulation of *PGRP-LC* or *Toll* upregulates AMPs, we overexpressed *PGRP-LCa, PGRP-LCx,* or *Toll* in S2 cells and examined the expression levels of AMPs. The overexpression of each protein upregulated *Mtk* (96.16±12.71, 218.72±10.08, and 3.04±0.72-fold for PGRP-LCa, PGRP-LCx, and Toll, respectively, *n*=3) and *DptB* (198.55±18.95, 376.74±5.33, and 2.28±0.3-fold for PGRP-LCa, PGRP-LCx, and Toll, respectively, *n*=3) (Fig. 3B,C). The expression levels of AMPs upon PGRP-LCa, PGRP-LCx, or Toll overexpression is summarized in Table 2. Other AMPs (*CecB*, *Dro*, and *Drs*) were also likewise upregulated when PGRP-LCa, PGRP-LCx, or Toll was overexpressed (Fig. S2). Notably, AMP production was induced more in cells overexpressing PGRP-LC than in those overexpressing Toll although the two proteins were overexpressed to a similar extent. In addition, *PGRP-LC* overexpression activated the expression of *Mtk*, which is a target of Toll. This result supports the crosstalk between the IMD and Toll pathways. Taken together, our observations indicate that SPS1 participates in the innate immune system by controlling the expression of the genes of two transmembrane receptors, *PGRP-LC* and *Toll*, and the amount of PGRP-LC affects the innate immune system more than the amount of Toll.

**Table 2.**
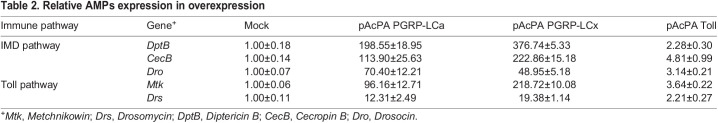
Relative AMPs expression in overexpression

## DISCUSSION

SPS1 is known to play an essential role in growth of cells, vitamin-B6 synthesis, and innate immunity in *D. melanogaster* (Shim et al., 2009; Lee et al., 2011). Among these various functions of SPS1, we focused on how SPS1 affects the immune system, especially on the AMP overproduction upon SPS1 deficiency. Via *Sps1* knockdown in S2 cells, we found that SPS1 regulates both the IMD and Toll pathways in the innate immune system.

In this study, we identified *PGRP-LC* as the primary target gene of the IMD pathway. *PGRP-LC* is known as the most upstream gene in the sub-cellular IMD pathway (Buchon et al., 2014). Recently, *PGRP-SD* was found to be involved in the IMD pathway outside the cell. It binds to peptidoglycans (PGNs) that are produced by digestion of bacterial cell walls, and the PGRP-SD/PGN complex helps re-localization of PGN to PGRP-LC on the cell surface (Iatsenko et al., 2016). Our results from the co-knockdown experiments clearly revealed that SPS1 targets PGRP-LC, not PGRP-SD, to induce the IMD pathway upon SPS1 deficiency. Toll was also found to be the primary target of SPS1 to induce the Toll pathway. The induction of both the IMD and Toll pathways is triggered by the upregulation of transmembrane receptors–PGRP-LC and Toll for the IMD and Toll pathways, respectively.

Two different lines of evidence support our findings that the induction of PGRP-LC and Toll pathway triggers the activation of AMP expression. First, knocking down SPS1 (SelD) induced *Diptericin* expression, whereas knocking down PGRP-LC inhibited *Diptericin* expression (Foley and O'Farrell, 2004). Second, the activation of PGRP-LC by ecdysone treatment upregulated AMPs (Rus et al., 2013). Altogether, our results indicate that upregulation of PGRP-LC is sufficient to induce the AMP signaling pathway. *Toll* overexpression has been reported to slightly induce the expression of *Drosomycin* (approximately 5-fold) (Hu et al., 2004). However, it is unclear why the upregulation of *Toll* had an insignificant effect on the activation of the innate immune pathways.

We found that the activation of AMP expression occurs as a result of a crosstalk between the IMD and Toll pathways. A crosstalk between these two innate immune pathways has been previously reported (Tanji et al., 2007; Nishide et al., 2019). Tanji et al. showed that overexpression of *PGRP-LC* or *Toll* activated both the IMD and Toll pathways. In our study, we also showed that overexpression of *PGRP-LC* or *Toll* induces both these pathways. In addition, our co-knockdown experiments showed that knockdown of *PGRP-LC* or *Toll* downregulates the AMPs in both the IMD and Toll pathways.

It was reported with microarray analysis that SPS1 deficiency upregulates the genes that participate in defense response, including *PGRP-SD*, *PGRP-LF*, and *pirk* (Lee et al., 2011). In this study, we found that PGRP-SD, unlike PGRP-LC, did not affect the activation of the innate-immune signaling induced upon SPS1 deficiency. Since *PGRP-SD* itself is a target gene of the IMD pathway and is activated upon PGRP-LC upregulation (Iatsenko et al., 2016), it is upregulated upon SPS1 deficiency presumably because the innate immune system is activated. PGRP-LF and Pirk suppress the IMD pathway and act as negative feedback regulators of this pathway (Maillet et al., 2008; Kleino et al., 2008). Therefore, the upregulation of *PGRP-LF* and *pirk* might be due to the upregulation of AMP genes upon SPS1 deficiency.

Recently, SPS1 has been suggested to regulate redox homeostasis (Tobe et al., 2016; Bang et al., 2021; Jung et al., 2021). To examine whether SPS1 affects the innate immune system through ROS in *D. melanogaster*, we knocked down *Sps1* in S2 cells and then treated the cells with the antioxidant N-acetyl cysteine (NAC) to reduce ROS. We found that the expression levels of *PGRP-LC* and *Toll* were not consequently changed, and this result suggests that the regulation of the innate immune system through SPS1 is independent of the redox system (Fig. S3A).

In the previous study, we showed that the vitamin B6 metabolism was regulated by the intracellular SPS1 levels in *Drosophila* S2 cells (Lee et al., 2011). We examined the effect of vitamin B6 on innate immunity in scrutiny in this study. When 4-deoxypyridoxine, an inhibitor of PLP biosynthesis, was administered to the cells without knockdown of *Sps1*, *PGRP-LC* and *Toll* were also upregulated, as in the case of *Sps1* knockdown (Fig. S3B). Therefore, it seems that SPS1 controls the expression of *PGRP-LC* and *Toll* by regulating the synthesis of vitamin B6. Like in *D. melanogaster*, the effects of vitamin B6 on immunity were also examined in a mouse model. The deficiency of vitamin B6 that was achieved by feeding the mice a low vitamin B6 diet led to increased immunoglobulin E production, presumably by the upregulation of interleukin-4 (Doke et al., 1997). The deficiency of vitamin B6, however, suppressed immunoglobulin G or immunoglobulin M (Kumar and Axelrod, 1968). Notably, Excess vitamin B6 levels affected immunity in the opposite manner (Inubushi et al. 2000). Therefore, it seems that vitamin B6 regulates immune homeostasis in mammalian systems. However, it remains unclear whether vitamin B6 also regulates immune homeostasis in *D. melanogaster*. An *in vivo* study may provide further insights into this issue.

A comparison of the mRNA levels of the AMPs induced upon *Sps1* knockdown with those induced upon immune stimulation would be interesting. An *in vivo* study may also facilitate evaluation of the detailed relationship *Sps1* knockdown and immune stimulation. Therefore, to elucidate the function of SPS1 in more detail and to confirm the findings of our current *in vitro* study, an *in vivo* study using a fruit fly system may be helpful. As shown by Alsina et al., SPS1-deficient fruit flies die in the late larval stage (Alsina et al., 1998). Thus, it would be intriguing to examine the effects of SPS1 deficiency on the expression of *PGRP-LC* and *Toll*, upregulation of AMPs, response upon immune stimulation, and the mechanism how vitamin B6 regulates the innate immunity using *Sps1*-knockout *D. melanogaster* larva.

Although our study has some pitfalls, we provide an important finding that SPS1 regulates the innate immune system of *D. melanogaster* by controlling the expression of *PGRP-LC* and *Toll* without any other immune stimulation.

## MATERIALS AND METHODS

### Materials

*Drosophila* Schneider cell line 2 (S2) was purchased from Invitrogen. HyQ SFX-Insect medium was purchased from Hyclone, T3 Megascript kit was purchased from Ambion, PowerUp™ SYBR™ Green Master Mix was purchased from Thermo Fisher, TRIzol reagent was purchased from Invitrogen, Moloney murine leukemia virus reverse transcriptase and nPfu forte DNA polymerase were purchased from Enzynomics. Dimethyldioctadecyl ammoniumbromide was purchased from Sigma-Aldrich, and oligonucleotides were purchased from Cosmo Genetech. The sequences of oligos used for RT-PCR and dsRNA are listed in Supplementary data.

### Vector Construction

pAcPA PGRP-LCa was produced by PCR amplification of *Bam*HI-PGRP-LCa-*Kpn*I from cDNA of S2 cells, cut and ligated into the pAcPA vector (Shim et al., 2009), containing the actin 5C promoter, with *Bam*HI/*Kpn*I. pAcPA PGRP-LCx and pAcPA Toll were prepared in the same way.

### Double-stranded RNA preparation *in vitro*

To prepare double-stranded RNA (dsRNA) of *Sps1*, *PGRP-SD*, *PGRP-LC*, *SPE* and *Toll*, each gene was amplified with a primer pair. The sequences of each primer are provided in Table S2. Each primer was fused with a T3 promoter sequence (5′-AATTAACCCTCACTAAAGGG-3′) at its 5′ end. *In vitro* transcription was performed using the T3 Megascript kit according to the manufacturer's protocols and then the dsRNAs were produced by annealing each complementary strand set.

### S2 cell culture and RNA interference

S2 cell culture and RNA interference using dsRNAs were carried out as described previously with minor modification (Shim et al., 2009). Briefly, for RNA interference, 2.5×10^5^ cells were plated on a 24-well plate containing 0.5 ml of HyQ SFX-Insect medium. Four micrograms of dsRNAs were added directly to the medium and incubated for 48 h and cells were split into appropriate culture dishes for further incubation and other experiments.

### RNA extraction and reverse transcription-quantitative PCR (RT-qPCR)

RT-qPCR was carried out as described with minor modification (Shim et al., 2009). Briefly, total RNA was isolated from the cells using the TRIzol reagent. cDNAs were synthesized from total RNAs with Moloney murine leukemia virus reverse transcriptase and oligo (dT) primers according to the manufacturer's protocols. RT-qPCR was carried out using an ABI 7300 real-time PCR system (Applied Biosystems) as follows. cDNAs were amplified using SYBR Green mix and specific primers for 40 cycles [initial incubation at 50°C for 2 min and then at 95°C for 10 min, and 40 cycles (95°C for 15 s, 55°C for 1 min, and 72°C for 1 min)]. Output data was obtained as Ct values using Sequence Detection Software (SDS) version 1.3 (7300 System, Applied Biosystems) and the differential mRNA expressions of each gene between control and knockdown cell were calculated using the comparative Ct method (Schmittgen and Livak, 2008). *rp49* mRNA, an internal control, was amplified along with the target genes, and the Ct value of *rp49* was used to normalize the expression of target genes.

### DNA transfection

Vectors were transfected into S2 cells as described previously (Han, 1996) with minor modifications. Briefly, 2 μg of pAcPA (backbone vector), pAcPA PGRP-LCa, pAcPA PGRP-LCx, and pAcPA Toll were mixed with 100 μl of dimethyldioctadecyl ammonium bromide (125 μg/ml) and 200 μl of HyQ-SFX-Insect media. The mixture was incubated for 30 min at room temperature and then added into a well of a six-well plate containing 2×10^6^ cells.

### Statistics

Each experiment was performed in biological triplicate for statistical analysis. Statistical analyses were performed using an unpaired Student's *t*-test or one-way ANOVA followed by Tukey's multiple comparison test. A value of *P*<0.05 was considered significant.

## Supplementary Material

Supplementary information

## References

[BIO059295C1] Allmang, C., Wurth, L. and Krol, A. (2009). The selenium to selenoprotein pathway in eukaryotes: more molecular partners than anticipated. *Biochim. Biophys. Acta* 1790, 1415-1423. 10.1016/j.bbagen.2009.03.003 19285539

[BIO059295C2] Alsina, B., Serras, F., Baguna, J. and Corominas, M. (1998). patufet, the gene encoding the Drosophila melanogaster homologue of selenophosphate synthetase, is involved in imaginal disc morphogenesis. *Mol. Gen. Genet.* 257, 113-123. 10.1007/s004380050630 9491069

[BIO059295C3] Bang, J., Han, M., Yoo, T. J., Qiao, L., Jung, J., Na, J., Carlson, B. A., Gladyshev, V. N., Hatfield, D. L., Kim, J. H. et al. (2021). Identification of signaling pathways for early embryonic lethality and developmental retardation in Sephs1(-/-) mice. *Int. J. Mol. Sci.* 22, 11647. 10.3390/ijms222111647 34769078 PMC8583877

[BIO059295C4] Brigelius-Flohe, R. (2008). Selenium compounds and selenoproteins in cancer. *Chem. Biodivers.* 5, 389-395. 10.1002/cbdv.200890039 18357548

[BIO059295C5] Brigelius-Flohé, R. and Sies, H. (2016). *Diversity of Selenium Functions in Health and Disease*. Boca Raton: CRC Press, Taylor & Francis Group.

[BIO059295C6] Buchon, N., Silverman, N. and Cherry, S. (2014). Immunity in Drosophila melanogaster - from microbial recognition to whole-organism physiology. *Nat. Rev. Immunol.* 14, 796-810. 10.1038/nri3763 25421701 PMC6190593

[BIO059295C7] Doke, S., Inagaki, N., Hayakawa, T. and Tsuge, H. (1997). Effect of vitamin B6 deficiency on an antibody production in mice. *Biosci. Biotechnol. Biochem.* 61, 1331-1336. 10.1271/bbb.61.1331 9301116

[BIO059295C8] Ehrenreich, A., Forchhammer, K., Tormay, P., Veprek, B. and Bock, A. (1992). Selenoprotein synthesis in E. coli. Purification and characterisation of the enzyme catalysing selenium activation. *Eur. J. Biochem.* 206, 767-773. 10.1111/j.1432-1033.1992.tb16983.x 1606960

[BIO059295C9] Foley, E. and O'Farrell, P. H. (2004). Functional dissection of an innate immune response by a genome-wide RNAi screen. *PLoS Biol.* 2, E203. 10.1371/journal.pbio.0020203 15221030 PMC434151

[BIO059295C10] Gladyshev, V. N., Hatfield, D. L., Schweizer, U. and Tsuji, P. A. (2016). *Selenium: Its Molecular Biology and Role in Human Health in pp. 1 online resource (XXXI, 628 pages 88 illustrations, 59 illustrations in Color*. Cham: Springer International Publishing: Imprint: Springer.

[BIO059295C11] Glass, R. S., Singh, W. P., Jung, W., Veres, Z., Scholz, T. D. and Stadtman, T. C. (1993). Monoselenophosphate: synthesis, characterization, and identity with the prokaryotic biological selenium donor, compound SePX. *Biochemistry* 32, 12555-12559. 10.1021/bi00210a001 8251472

[BIO059295C12] Guimaraes, M. J., Peterson, D., Vicari, A., Cocks, B. G., Copeland, N. G., Gilbert, D. J., Jenkins, N. A., Ferrick, D. A., Kastelein, R. A., Bazan, J. F. et al. (1996). Identification of a novel selD homolog from eukaryotes, bacteria, and archaea: is there an autoregulatory mechanism in selenocysteine metabolism? *Proc. Natl. Acad. Sci. USA* 93, 15086-15091. 10.1073/pnas.93.26.15086 8986768 PMC26360

[BIO059295C13] Han, K. H. (1996). An efficient DDAB-mediated transfection of Drosophila S2 cells. *Nucleic Acids Res.* 24, 4362-4363. 10.1093/nar/24.21.4362 8932397 PMC146234

[BIO059295C14] Hatfield, D. L. and Gladyshev, V. N. (2002). How selenium has altered our understanding of the genetic code. *Mol. Cell. Biol.* 22, 3565-3576. 10.1128/MCB.22.11.3565-3576.2002 11997494 PMC133838

[BIO059295C15] Hu, X., Yagi, Y., Tanji, T., Zhou, S. and Ip, Y. T. (2004). Multimerization and interaction of toll and Spatzle in Drosophila. *Proc. Natl. Acad. Sci. USA* 101, 9369-9374. 10.1073/pnas.0307062101 15197269 PMC438983

[BIO059295C16] Iatsenko, I., Kondo, S., Mengin-Lecreulx, D. and Lemaitre, B. (2016). PGRP-SD, an extracellular pattern-recognition receptor, enhances peptidoglycan-mediated activation of the Drosophila Imd pathway. *Immunity* 45, 1013-1023. 10.1016/j.immuni.2016.10.029 27851910

[BIO059295C17] Inubushi, T., Okada, M., Matsui, A., Hanba, J., Murata, E. and Katunuma, N. (2000). Effect of dietary vitamin B6 contents on antibody production. *Biofactors* 11, 93-96. 10.1002/biof.5520110127 10705972

[BIO059295C18] Janeway, C. A., Jr. and Medzhitov, R. (2002). Innate immune recognition. *Annu. Rev. Immunol.* 20, 197-216. 10.1146/annurev.immunol.20.083001.084359 11861602

[BIO059295C19] Jung, J., Kim, Y., Na, J., Qiao, L., Bang, J., Kwon, D., Yoo, T. J., Kang, D., Kim, L. K., Carlson, B. A. et al. (2021). Constitutive oxidative stress by SEPHS1 deficiency induces endothelial cell dysfunction. *Int. J. Mol. Sci.* 22, 11646. 10.3390/ijms222111646 34769076 PMC8584027

[BIO059295C20] Kanoh, H., Tong, L. L., Kuraishi, T., Suda, Y., Momiuchi, Y., Shishido, F. and Kurata, S. (2015). Genome-wide RNAi screening implicates the E3 ubiquitin ligase Sherpa in mediating innate immune signaling by Toll in Drosophila adults. *Sci. Signal.* 8, ra107. 10.1126/scisignal.2005971 26508789

[BIO059295C21] Kleino, A. and Silverman, N. (2014). The Drosophila IMD pathway in the activation of the humoral immune response. *Dev. Comp. Immunol.* 42, 25-35. 10.1016/j.dci.2013.05.014 23721820 PMC3808521

[BIO059295C22] Kleino, A., Myllymaki, H., Kallio, J., Vanha-aho, L. M., Oksanen, K., Ulvila, J., Hultmark, D., Valanne, S. and Ramet, M. (2008). Pirk is a negative regulator of the Drosophila Imd pathway. *J. Immunol.* 180, 5413-5422. 10.4049/jimmunol.180.8.5413 18390723

[BIO059295C23] Kumar, M. and Axelrod, A. E. (1968). Cellular antibody synthesis in vitamin B6-deficient rats. *J. Nutr.* 96, 53-59. 10.1093/jn/96.1.53

[BIO059295C24] Lee, B. J., Worland, P. J., Davis, J. N., Stadtman, T. C. and Hatfield, D. L. (1989). Identification of a selenocysteyl-tRNA(Ser) in mammalian cells that recognizes the nonsense codon, UGA. *J. Biol. Chem.* 264, 9724-9727. 10.1016/S0021-9258(18)81714-8 2498338

[BIO059295C25] Lee, K. H., Shim, M. S., Kim, J. Y., Jung, H. K., Lee, E., Carlson, B. A., Xu, X. M., Park, J. M., Hatfield, D. L., Park, T. et al. (2011). Drosophila selenophosphate synthetase 1 regulates vitamin B6 metabolism: prediction and confirmation. *BMC Genomics* 12, 426. 10.1186/1471-2164-12-426 21864351 PMC3218224

[BIO059295C26] Leinfelder, W., Stadtman, T. C. and Bock, A. (1989). Occurrence in vivo of selenocysteyl-tRNA(SERUCA) in Escherichia coli. Effect of sel mutations. *J. Biol. Chem.* 264, 9720-9723. 10.1016/S0021-9258(18)81713-6 2524495

[BIO059295C27] Lemaitre, B. and Hoffmann, J. (2007). The host defense of Drosophila melanogaster. *Annu. Rev. Immunol.* 25, 697-743. 10.1146/annurev.immunol.25.022106.141615 17201680

[BIO059295C28] Lindsay, S. A. and Wasserman, S. A. (2014). Conventional and non-conventional Drosophila Toll signaling. *Dev. Comp. Immunol.* 42, 16-24. 10.1016/j.dci.2013.04.011 23632253 PMC3787077

[BIO059295C29] Longtin, R. (2004). A forgotten debate: is selenocysteine the 21st amino acid? *J. Natl. Cancer Inst.* 96, 504-505. 10.1093/jnci/96.7.504 15069108

[BIO059295C30] Low, S. C., Harney, J. W. and Berry, M. J. (1995). Cloning and functional characterization of human selenophosphate synthetase, an essential component of selenoprotein synthesis. *J. Biol. Chem.* 270, 21659-21664. 10.1074/jbc.270.37.21659 7665581

[BIO059295C31] Lu, J. and Holmgren, A. (2009). Selenoproteins. *J. Biol. Chem.* 284, 723-727. 10.1074/jbc.R800045200 18757362

[BIO059295C32] Maillet, F., Bischoff, V., Vignal, C., Hoffmann, J. and Royet, J. (2008). The Drosophila peptidoglycan recognition protein PGRP-LF blocks PGRP-LC and IMD/JNK pathway activation. *Cell Host Microbe* 3, 293-303. 10.1016/j.chom.2008.04.002 18474356

[BIO059295C33] Myllymaki, H., Valanne, S. and Ramet, M. (2014). The Drosophila imd signaling pathway. *J. Immunol.* 192, 3455-3462. 10.4049/jimmunol.1303309 24706930

[BIO059295C34] Na, J., Jung, J., Bang, J., Lu, Q., Carlson, B. A., Guo, X., Gladyshev, V. N., Kim, J., Hatfield, D. L. and Lee, B. J. (2018). Selenophosphate synthetase 1 and its role in redox homeostasis, defense and proliferation. *Free Radic. Biol. Med.* 127, 190-197. 10.1016/j.freeradbiomed.2018.04.577 29715549

[BIO059295C35] Nishide, Y., Kageyama, D., Yokoi, K., Jouraku, A., Tanaka, H., Futahashi, R. and Fukatsu, T. (2019). Functional crosstalk across IMD and Toll pathways: insight into the evolution of incomplete immune cascades. *Proc R Soc B Biol Sci* 286, 20182207. 10.1098/rspb.2018.2207 PMC6408883 30963836

[BIO059295C36] Paquette, N., Broemer, M., Aggarwal, K., Chen, L., Husson, M., Erturk-Hasdemir, D., Reichhart, J. M., Meier, P. and Silverman, N. (2010). Caspase-mediated cleavage, IAP binding, and ubiquitination: linking three mechanisms crucial for Drosophila NF-kappaB signaling. *Mol. Cell* 37, 172-182. 10.1016/j.molcel.2009.12.036 20122400 PMC2819219

[BIO059295C37] Rus, F., Flatt, T., Tong, M., Aggarwal, K., Okuda, K., Kleino, A., Yates, E., Tatar, M. and Silverman, N. (2013). Ecdysone triggered PGRP-LC expression controls Drosophila innate immunity. *EMBO J.* 32, 1626-1638. 10.1038/emboj.2013.100 23652443 PMC3671248

[BIO059295C38] Schmittgen, T. D. and Livak, K. J. (2008). Analyzing real-time PCR data by the comparative C-T method. *Nat. Protoc.* 3, 1101-1108. 10.1038/nprot.2008.73 18546601

[BIO059295C39] Shim, M. S., Kim, J. Y., Jung, H. K., Lee, K. H., Xu, X. M., Carlson, B. A., Kim, K. W., Kim, I. Y., Hatfield, D. L. and Lee, B. J. (2009). Elevation of glutamine level by selenophosphate synthetase 1 knockdown induces megamitochondrial formation in Drosophila cells. *J. Biol. Chem.* 284, 32881-32894. 10.1074/jbc.M109.026492 19755423 PMC2781704

[BIO059295C40] Squires, J. E. and Berry, M. J. (2008). Eukaryotic selenoprotein synthesis: mechanistic insight incorporating new factors and new functions for old factors. *IUBMB Life* 60, 232-235. 10.1002/iub.38 18344183

[BIO059295C41] Tanji, T., Hu, X. D., Weber, A. N. R. and Ip, Y. T. (2007). Toll and IMD pathways synergistically activate an innate immune response in Drosophila melanogaster. *Mol. Cell. Biol.* 27, 4578-4588. 10.1128/MCB.01814-06 17438142 PMC1900069

[BIO059295C42] Tobe, R., Carlson, B. A., Huh, J. H., Castro, N. P., Xu, X. M., Tsuji, P. A., Lee, S. G., Bang, J., Na, J. W., Kong, Y. Y. et al. (2016). Selenophosphate synthetase 1 is an essential protein with roles in regulation of redox homoeostasis in mammals. *Biochem. J.* 473, 2141-2154. 10.1042/BCJ20160393 27208177 PMC5094348

[BIO059295C43] Valanne, S., Wang, J. H. and Ramet, M. (2011). The Drosophila Toll signaling pathway. *J. Immunol.* 186, 649-656. 10.4049/jimmunol.1002302 21209287

[BIO059295C44] Xu, X. M., Carlson, B. A., Mix, H., Zhang, Y., Saira, K., Glass, R. S., Berry, M. J., Gladyshev, V. N. and Hatfield, D. L. (2007). Biosynthesis of selenocysteine on its tRNA in eukaryotes. *PLoS Biol.* 5, e4. 10.1371/journal.pbio.0050004 17194211 PMC1717018

[BIO059295C45] Zhang, L. J. and Gallo, R. L. (2016). Antimicrobial peptides. *Curr. Biol.* 26, R14-R19. 10.1016/j.cub.2015.11.017 26766224

