# Supplementary material for: SPS1 deficiency-triggered PGRP-LC and Toll expression controls innate immunity in Drosophila S2 cells
Source: Biol Open. 2022 Aug 1;11(8):bio059295. doi: 10.1242/bio.059295 (PMC9364239; doi:10.1242/bio.059295)
Supplement: Supplementary information [file biolopen-11-059295-s1.pdf]

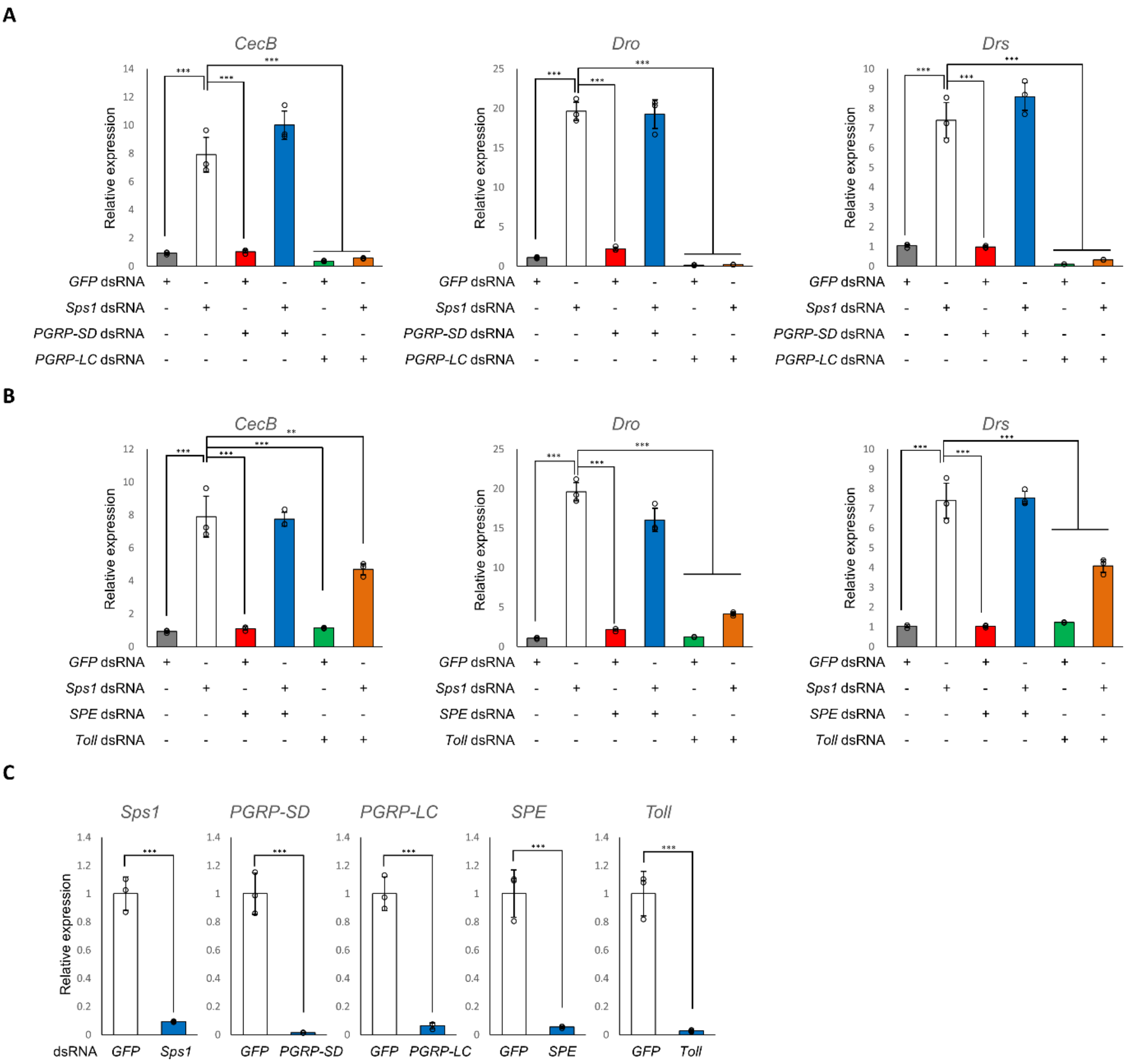

Fig. S1. Five days after adding *PGRP-SD*, *PGRP-LC*, *SPE*, and *Toll* dsRNA with *Sps1* dsRNA, the mRNA levels of AMP genes were measured by RT-qPCR using rp49 as a control for normalization. The gene symbol is shown in each graph. Relative expression level of AMP genes after double knockdown of *SPS1* with *PGRP-SD* and *PGRP-LC* (A), *SPS1* with *SPE* and *Toll* (B). Knockdown efficiency of *SPS1*, *PGRP-SD*, *PGRP-LC*, *SPE*, and *Toll* (C). **, *** indicates p-value < 0.01, 0.001, respectively. as determined by one-way ANOVA with Tukey's multiple comparison test. *CecB*, *Cecropin B*; *Dro*, *Drosocin*; *Drs*, *Drosomycin*; *SPE*, spätzle-processing enzyme.

Biology Open • Supplementary information

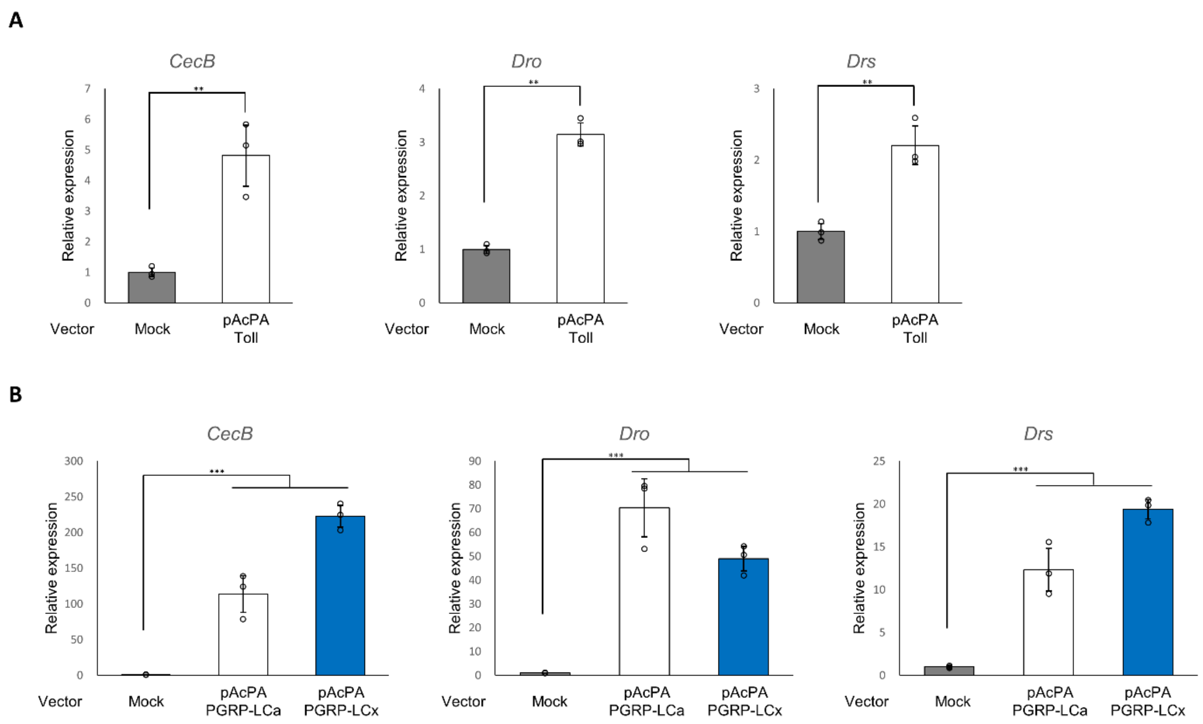

Fig. S2. (A, B) Three days after transfection of pAcPA PGRP-LCa, pAcPA PGRP-LCx, and pAcPA Toll, the mRNA levels of AMP genes were measured by RT-qPCR using rp49 as a control for normalization. **, *** indicates p-value < 0.01, 0.001, respectively. P-value was calculated using unpaired Student's t-test. *CecB*, *Cecropin B*; *Dro*, *Drosocin*; *Drs*, *Drosomycin*.

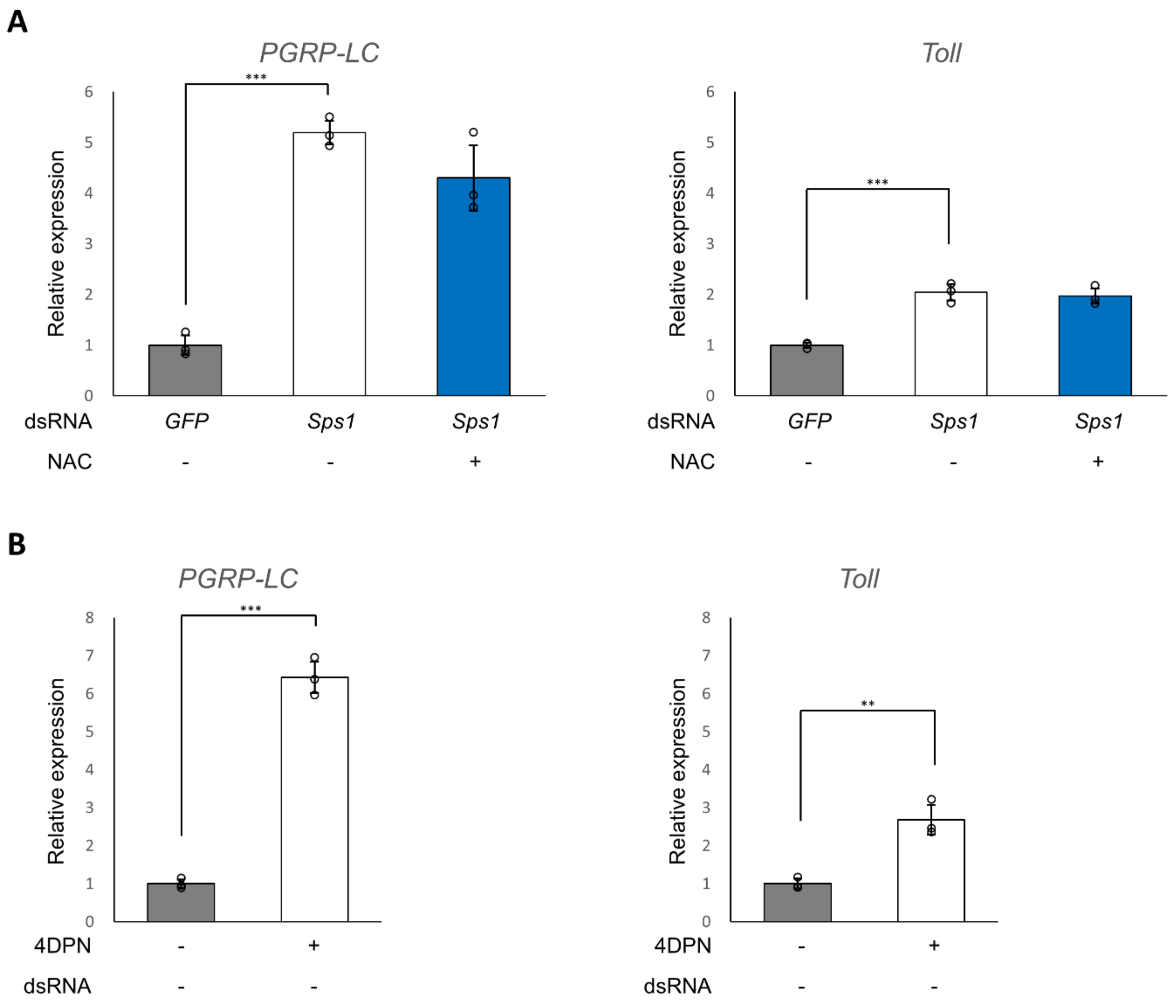

Fig. S3. (A) Five days after adding *Sps1* dsRNA and 3mM NAC, the mRNA levels of PGRP-LC and Toll were measured by RT-qPCR. (B) four days after treatment of 60 µg/ml of 4DPN, the mRNA levels of PGRP-LC and Toll were measured by RT-qPCR. Rp49 was used as a control for normalization. **, *** indicates p-value < 0.01, 0.001, respectively. P-value was calculated using unpaired Student's t-test.

Biology Open • Supplementary information

Table S1. List of primers for qRT-PCR

Gene	Sequence（5'→3'）	
	Forward	Reverse
RP49	CAGTCGGATCGATATGCTA	AATCTCCTTGCGCTTCTT
DptB	ATCCTGATCCCCGAGAGATT	TGAAGTGCCCTAAAACCTGAA
CecB	AATCCGATCGTAAGCCAACA	AGAGAAATGAGCGGGTCGAG
Dro	CATACCGCGGAGAAGTCATC	TTAGGGGACAAACCCATTCA
Mtk	CCACCGAGCTAAGATGCAA	TGTTAACGACATCAGCAGTGTG
Drs	GTACTTGTTCGCCCTCTTCG	ACAGGTCTCGTTGTCCCAGA
SPS1	AGGGGATGTACTGGTGCTAA	TCTTATTGCCCTTCTCAACG
PGRP-SD	CCTTTGCCGGTCCCAATAAC	GGAGTGGTCTTTACGTGTCG
PGRP-LC	AGGGTCTAACGGTGATCAGT	AACTGTTGTATCGTCACGGG
SPE	CCAATATGCCTTCCCACGGA	TCCAATCGATGAAAGCCCCC
Toll	GACCAGAGCTTCATTGAGGA	AATCGCAGCTTATCCCAGAA

Biology Open • Supplementary information

Table S2. List of primers for dsRNA synthesis

Gene	Sequence（5'→3'）	
	Forward	Reverse
SPS1	AATTAACCCTCACTAAAGGGATGAG CTACGCCGCTGATG	AATTAACCCTCACTAAAGGGAGTT CATCGCCCGGTGGTA
PGRP-SD	AATTAACCCTCACTAAAGGGGAATG ACTTGGATCGGTTTGCT	AATTAACCCTCACTAAAGGGCTCC AGGACTCTTGGTAGCAC
PGRP-LC [-exon E]	AATTAACCCTCACTAAAGGGGTCAG CCGTTTTGCCATACG	AATTAACCCTCACTAAAGGGGTGA ACTGTTGGGCGGGTAG
PGRP-LC [exon E]	AATTAACCCTCACTAAAGGGAACTG TCTGAATGGGGTGCA	AATTAACCCTCACTAAAGGGCCCA CGAGAAAGTTGTAGCCT
SPE	AATTAACCCTCACTAAAGGGGGGTT GAGGGCTTGGTGAAT	AATTAACCCTCACTAAAGGGCGCA CCAACGCAATGTCATT
Toll	AATTAACCCTCACTAAAGGGACTCT TTGCACATACCACCA	AATTAACCCTCACTAAAGGGTGCGT AATTTGAACTGCCTG

Biology Open • Supplementary information
